# Clinical characterisation of women with persistent genital arousal disorder: the iPGAD-study

**DOI:** 10.1038/s41598-023-48790-2

**Published:** 2023-12-20

**Authors:** Franziska M. L. M. Kümpers, Christopher Sinke, Cordula Schippert, Katja Kollewe, Sonja Körner, Peter Raab, Bernhard Meyer, Sabine Maschke, Matthias Karst, Christian Sperling, Eleni Dalkeranidis, Tillmann H. C. Krüger

**Affiliations:** 1 Divison of Clinical Psychology and Sexual Medicine, Department of Psychiatry, Social Psychiatry and Psychotherapy, Hannover Medical School, Hannover, Germany; 2 Department of Obstetrics and Gynaecology, Hannover Medical School, Hannover, Germany; 3 Department of Neurology, Hannover Medical School, Hannover, Germany; 4 Department of Diagnostic and Interventional Neuroradiology, Hannover Medical School, Hannover, Germany; 5 Department of Diagnostic and Interventional Radiology, Hannover Medical School, Hannover, Germany; 6 Department of Anaesthesiology, Pain Clinic, Hannover Medical School, Hannover, Germany; 7 Center for Systems Neuroscience, Hannover, Germany

**Keywords:** Psychiatric disorders, Sexual dysfunction

## Abstract

Persistent Genital Arousal Disorder (PGAD) is a rare condition—mostly in women—where patients perceive prolonged genital arousal without any sexual desire or stimulation. Etiopathological considerations reach from peripheral to central issues over local disturbance of the pudendal nerve to neuropathy, psychosocial, and pharmacological theories. Since well controlled clinical studies about PGAD in conjunction with a mental and somatic health status are missing, this study is a detailed clinical investigation of PGAD patients compared to healthy controls. 26 women who fulfilled diagnostic criteria for PGAD were compared to 26 age matched healthy controls. Investigations included comparison of vegetative, gynaecological and sexual history, psychiatric features as well as a (neuro-)radiological, neurophysiological and gynaecological examination. Moreover, a detailed clinical characterisation of PGAD symptoms was performed. PGAD symptoms were mostly characterised as tingling or prickling and were permanently present. In over 80%, PGAD symptoms were located in the clitoris. Almost 70% reported radiations to other regions of the body. Most frequent trigger factors were tight clothes, mental stress, driving a car/bus/bicycle and sexual intercourse. Relieving factors were mainly distraction, relaxation, physical exercise, masturbation and swimming. In group comparisons, PGAD presented with significant higher rates of sexual dysfunctions, spontaneous orgasms, swelling of the genitals, extraordinary lubrication as well as higher rates in depression, agoraphobia, generalized anxiety disorder and lifetime panic disorder. Significantly more PGAD patients were diagnosed with restless legs symptoms. In contrast childhood traumatization, somatization disorder, suicidality, gynaecological as well as neurophysiological examination of the pudendal nerve were not different between the groups. MRI of the brain, pelvis and spinal cord was unsuspicious and incidental findings - including Tarlov cysts or pelvic venous congestion - were equally distributed among the groups. In summary, our study provides a careful characterization of women with PGAD highlighting a serious mental burden, most probably as a consequence of PGAD. With the current set of clinical investigations there was no evidence of a clear causal relationship to a specific clinical finding as it has been previously discussed. Future studies and additional techniques will have to further explore where and how in the peripheral or central nervous systems PGAD develops.

## Introduction

Persistent genital arousal disorder (PGAD) is an apparently rare condition, where patients perceive prolonged genital arousal without any sexual desire. In PGAD, this sensation occurs despite the absence of a sexual stimulus and usually lasts for days or weeks and does not subside after one or more orgasms^1^. Primarily, PGAD is observed in women, with only a few reports on children or men^2–6^. Leiblum^7^ described five diagnostic criteria. More recent definitions by the International Society for the Study of Women’s Sexual Health also cover the criteria (for further details see methods—subjects and procedure)^8,9^. Valid data on the prevalence of PGAD is not available yet. Estimations go from 0.5 to 6.7%^5,8,10–14^. PGAD patients often feel ashamed and are afraid of being diagnosed with hypersexuality^10^ or are not appropriately examined at all.

There are some theories on the causes of PGAD pointing towards disturbances of the peripheral and/or central nervous system. Regarding peripheral factors compression of the dorsal branch of the pudendal nerve, e.g. by a periclitoral mass^10,12,15^, dorsal nerve injury due to pelvic trauma by e.g. horseback riding accidents^16^, bicycle riding^17^, birth injury or surgical trauma^18^ are discussed. The pudendal nerve not only transmits the perception of stimulation from the clitoris with the branch of the dorsal clitoral nerve, which is then interpreted as a sensation of arousal, but also transmits the perceptions of the areas of the perineal and posterior labial nerves^19^. Hence, nerve compression or injury shall produce the symptoms of continuous arousal^18^. Small fibre neuropathy has also been discussed as a cause of PGAD and the frequent intolerance towards tight clothing and prolonged sitting as a trigger of PGAD may support this assumption^18,20,21^. However, respective (histo-)pathological findings have not yet been reported^6,12,22^. Oaklander et al.^23^ suspect a connection to sensory polyneuropathy. Other theories suggest an overlap between PGAD, Restless Legs Syndrome (RLS) and Overactive Bladder Syndrome (OAB)^20,24^ and assume a dysregulation of neurovegetative afferent and efferent signals, supposing PGAD as a phenotypic variant of RLS and OAB representing the same pathologic state only in different areas^25–27^. Contradicting this assumption reports on sustained symptom release by administration of dopaminergic or anticholinergic drugs are missing. From a still peripheral, vascular position, pelvic varices or pelvic congestion syndrome are discussed factors associated with PGAD^20,22^.

With regard to the central nervous system, sacral meningeal cysts (e.g. Tarlov cysts)^8,12,20^ and intervertebral disc pathologies are discussed as possible causes of PGAD^8^. Other discussed neurological pathologies include epileptic foci^28^, sexual epileptic auras^29^, arteriovenous malformation^30^ and arteriovenous fistulas and strokes^31^. On a neuropsychopharmacological level there is some evidence that antidepressants, antipsychotics and anticonvulsants can both induce symptoms of PGAD during administration or (probably more often) discontinuation of the drug or even alleviate symptoms^32,33^. Theories have been proposed that improvement may be due to inhibition of sexual perception and induction of symptoms by disinhibition induced by drugs with a primarily serotonergic function^34–38^.

Finally, psychosocial factors may be relevant as possible causes or as comorbidities of PGAD ranging from stress to anxiety, panic, depression and obsessive compulsive disorder^8,14,20,25,39–41^ and even suicidal ideations may come along with PGAD^10,40,42,43^. Also, PGAD as a result of sexual abuse has been considered^39,44–46^.

In summary, many assumptions on possible causes and triggering factors of PGAD have been made, however, there is a lack of well controlled clinical studies (case control studies) incorporating a thorough clinical assessment of subjects with PGAD compared to age matched healthy controls. Researchers, clinicians and patients agree, that PGAD is a severely distressing and life-impairing disease, which requires intensified research based on systematic, controlled trials^8^. Based on previous research and above-mentioned possible causes of PGAD, Goldstein et al.^8^ proposed a systematic investigation of PGAD patients. The investigation should include the following 5 regions: (1) end organ, (2) pelvis and perineum, (3) cauda equina, (4) spinal cord, (5) brain. In addition, a detailed history should be taken, including the exact symptomatology, trigger and relieving factors, mental health and medication history. Hence, we initiated a clinical investigation aiming at systematically elucidating the different clinical levels and components that might contribute to PGAD using a well-controlled comprehensive multimodal assessment of PGAD patients and controls. Goldstein et al.^8^ also used the term of genito-pelvic dysesthesia (GPD). In this manuscript the consistently use of the term PGAD was agreed.

## Methods

### Subjects and procedure

**Figure 1:**
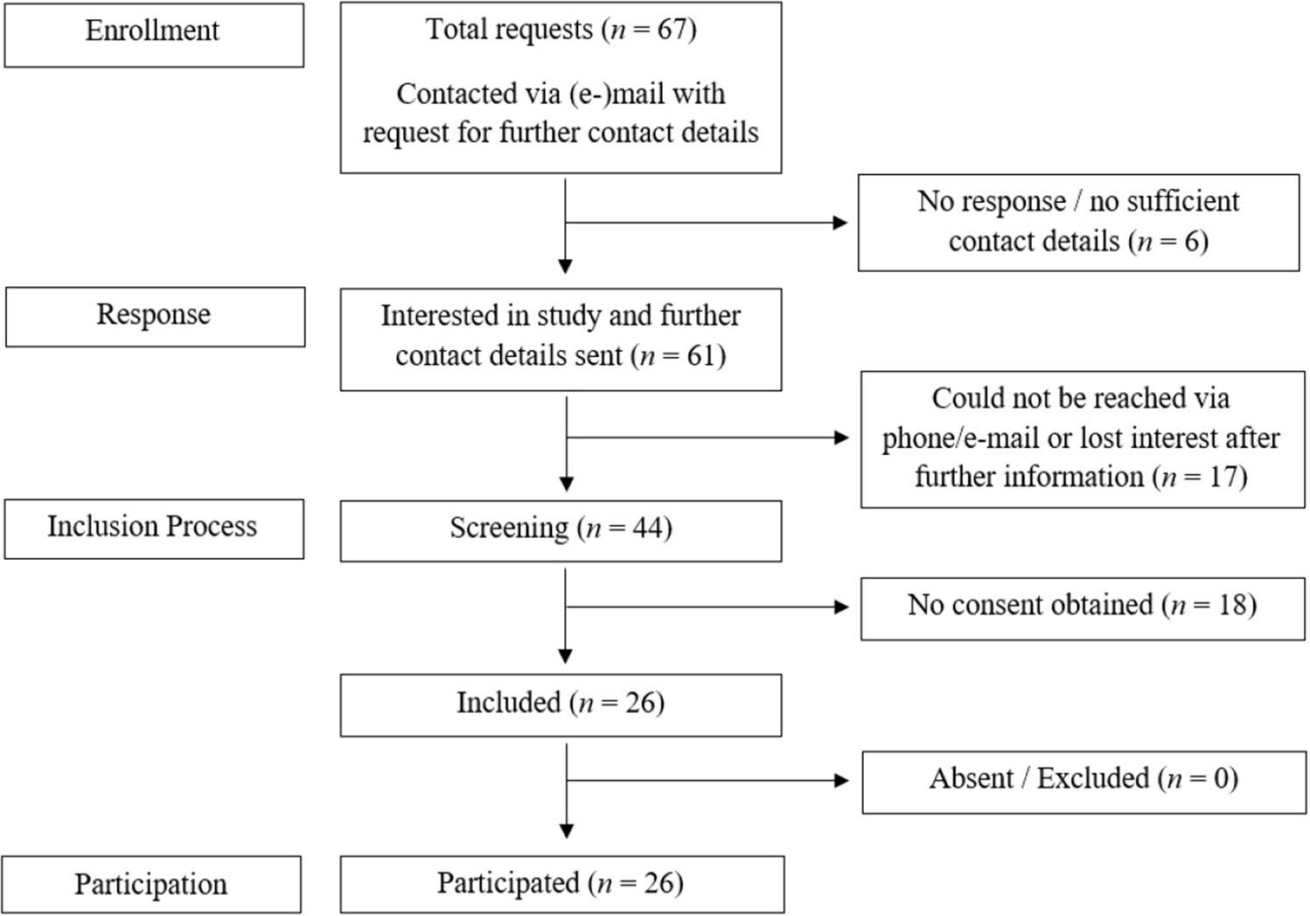
Recruitment of the PGAD group.

**Figure 2:**
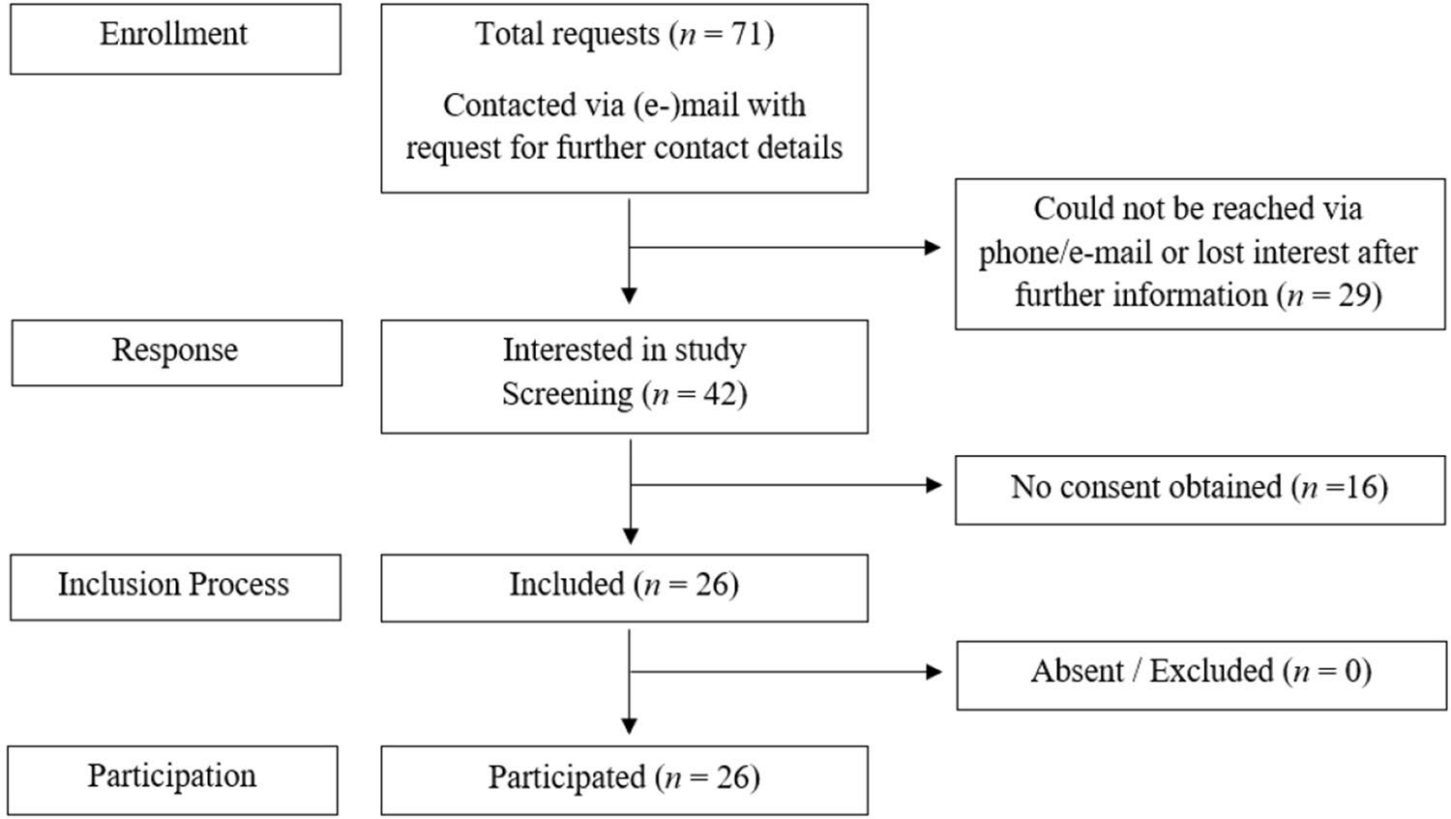
Recruitment of healthy control group.

A total of 52 subjects participated in the iPGAD study (Identification of Etiopathological and Clinical Factors in Persistent Genital Arousal Disorder: the iPGAD-Study), 26 patients suffering from PGAD and 26 control subjects. Data acquisition took place from June 2020 to August 2021. Subjects gave written informed consent to participate, were free to withdraw from the study at any time and received reimbursement for their participations. No subjects were excluded from the analyses (see Figs. 1 And 2). Patients suffering from PGAD were recruited via the sexual medicine consultation at Hannover Medical School or via social networks (in particular www.facebook.com and German internet forums for PGAD). The control subjects were recruited via the intranet of Hannover Medical School, social networks, and word of mouth. Control subjects were matched for age and years of education. This study was conducted in accordance with the Declaration of Helsinki 1964, updated in October 2013 and was approved by the Ethics Committee of Hannover Medical School (No. 8589_BO_S_2019). The study was registered at ClinicalTrials.gov (Protocol ID: 8589_BO_S_2019; NCT04566783).

Inclusion criteria for control subjects were defined as follows: female gender, age between 18 and 75 years, proficiency in German writing and language, no acute and severe mental or somatic disease requiring immediate treatment. In addition, PGAD patients should fulfil the criteria of Leiblum and Nathan^47^. These include: (1) Prolonged persistence of involuntary genital and clitoral arousal over an extended period of time (hours, days, or months), (2) no subsiding of genital arousal despite one or more orgasms, (3) no association of physical genital arousal with subjective feelings of sexual arousal or sexual desire, (4) the arousal is not only triggered by sexual activity, but also by non-sexual stimuli, or without any obvious triggers, (5) the arousal is experienced as unwanted and intrusive; correspondingly, distress is associated with it. A severe intelligence impairment, acute physical or mental illness (e.g. acute psychosis, brain damage, Alzheimer’s disease, severe bacterial infection) and contraindication to magnetic resonance imaging were the exclusion criteria for both groups.

### Measures

Variables for the following four domains were assessed: (1) sociodemographic data and neurodevelopmental factors, (2) clinical and phenomenological characterisation of PGAD symptoms including sexual characteristics, (3) psychological features including psychiatric comorbidities and personality traits, (4) apparative diagnostic measures.

### Sociodemographic data and neurodevelopmental factors

In a semi-structured interview, sociodemographic data including age, highest educational qualification converted into years of education, employment status (employed, in training, unemployed, retired), body size and weight (Body Mass Index), handedness (left- or right-handed), the use or abuse of alcohol, nicotine and recreational drugs, medication, marital status (unmarried or single, married, divorced) and the number of births, miscarriages and abortions were collected.

### Vegetative, gynaecological and sexual history and characterisation of PGAD

#### Gynaecology and vegetative history

In the context of a semi-structured interview, a survey of the gynaecological history including micturition (especially dysuria, urinary urgency and frequency), the duration of the menstrual cycle (excluding those who were menopausal or stopped menstruating due to hormonal contraception), dysmenorrhea or eumenorrhea, menopause, menarche, masturbarche, contraception and infertility treatment was taken.

#### Sexual history

The sexual orientation was accessed based on the Kinsey Scale^48^. The categories were modified to heterosexual, bisexual and homosexual. Hypersexual disorder was assessed using the four Kafka criteria^49^. Furthermore, the subjects were specifically asked for spontaneous orgasms, swelling of the genitals and an extraordinary lubrication.

#### Characterization of PGAD

In addition, the detailed history of the PGAD symptomatology was taken. The age at first symptomatology and the duration of the disease were assessed. PGAD patients were asked for trigger and relief factors and their medication concerning the PGAD symptoms. To better map the PGAD symptomatology, a specific questionnaire was developed based on the pain detect questionnaire^50^. Symptom severity was assessed at three different time points (now, most severe symptoms in the last 4 weeks, average symptom severity in the last 4 weeks) using visual analogue scale (VAS) from 0 to 10. The variables surveyed also included the course of the symptoms (continuous symptoms with slight fluctuations, continuous symptoms with symptom attacks, symptom attacks and symptom-free phases in between, symptom attacks and symptoms in between). The quality of the symptoms (burning, tingling or prickling, touch, lightning-like or electrifying, cold, heat/warmth, numbness, slight pressure) was measured by Likert-type questions (never, hardly, slight, moderate, strong, very strong). It was also assessed, whether there is radiation to other regions of the body (e.g., abdomen, back, legs, arms, breasts). To specify the exact localisation of the symptomatology, the patient could mark the concerned regions on a body illustration. They could set as many crosses as they wanted (mons pubis, urethra, vaginal opening, pubic bone, clitoris, inner and outer labia, anus, uterine region, ovarian region).

### Psychological features including psychiatric comorbidities and personality

In a semi-structured interview, it was assessed whether ICD-10 criteria for sexual dysfunction (ICD10 F52, including lack or loss of sexual desire, sexual aversion, lack of sexual satisfaction, failure of genital responses, orgasmic disturbances, nonorganic vaginismus, non-organic dyspareunia and increased sexual desire) and somatization disorder (ICD-10 F45, including undifferentiated somatization disorder, hypochondriacal disorder, somatoform autonomic dysfunction, persistent somatoform pain disorder, other and unspecified somatoform disorder) were met. A possible somatoform pain disorder was measured by using the German Screening for Somatoform Disorders (SOMS^51^). Psychiatric comorbidities were diagnosed using the German version of the International Neuropsychiatric Interview for Axis I Disorders (MINI^52^). Comorbid personality disorders were assessed via the German version of the Structured Clinical Interview for DSM-IV Axis II Disorders (SKID II^53^). Anxiety and Depression were assessed using the Hospital Anxiety and Depression Scale (HADS^54^). The quality of sleep was measured by means of the Pittsburgh Sleep Quality Index (PSQI^55^). This includes the variables sleep quality, sleep latency, sleep duration, sleep efficiency, sleep disturbance, the use of sleeping pills and daytime sleepiness. Quality of life was measured using the short form of WHO-Quality of Life^56^. The history of childhood sexual abuse was evaluated via the German version of the Childhood Trauma Questionnaire (CTQ^57^), which is used to screen for histories of five types of maltreatment during childhood (physical, emotional and sexual abuse as well as a physical and emotional neglect).

### (Neuro-)radiological, neurophysiological and gynaecological examination

All subjects received a neurological clinical examination. The neurophysiological examination consisted of electroneurography of peripheral nerves as well as somatosensory evoked potentials (SEP) of the pudendal and tibial nerve. Latencies and amplitudes were measured in SEP. The electroneurography of peripheral nerves included tibial, peroneal, sural, median or ulnar nerve. Nerve conduction velocity (motoric or sensitive), distal motor latency, amplitudes and F-waves were recorded and analysed.

In addition, all subjects received a gynaecological examination. A speculum examination and palpation were performed. Also, a sonographic assessment of the ovary, uterus and endometrium was undertaken. Examination was also performed for varices, free fluid, and possible other abnormalities. The overall impression of the gynaecological examination was assessed by a consultant in gynaecology.

An MRI of the head, pelvis and spinal cord from conus medullaris to sacral spine was performed on a 3T-MR scanner (Magnetom Skyra, Siemens, Germany) to determine possible organic causes (for imaging parameters see supplement 1). Images were interpreted by a consultant of neuroradiology and radiology, respectively.

### Data Analysis

All statistical analysis were conducted using SPSS Statistics Version 27 (IBM Corporation, Amonk, NY, USA). Descriptive statistics were calculated and are reported as mean (*M*) and standard deviation (± /SD). Data were checked for normal distribution using a Kolmogorow-Smirnow test. Analyses were carried out using independent t-tests and Fisher’s exact tests for dichotomous variables. As this was the first controlled study to distinguish differences between patients with PGAD and healthy controls regarding the set of clinical variables tested here, an exploratory approach was chosen and two-tailed significance levels without correction for multiple comparisons are reported (all analyses *p* < 0.05). Levene’s Test was used to verify the assumption of variance homogeneity.

## Results

### Sociodemographic data and neurodevelopmental factors

As intended by subject matching there were no group differences in the sociodemographic variables regarding age (*M*_*PGAD*_ = 39.5 ± 14.7; *M*_*Controls*_ = 39.5 ± 14.2; *p* = 1.00) and years of education (*M*_*PGAD*_ = 11.9 ± 1.4; *M*_*Controls*_ = 12.1 ± 1.4; *p* = 0.70). Also, employment status (*p* = 0.49), marital status (*p* = 0.72), Body Mass Index (*M*_*PGAD*_ = 23.5 ± 4.4, *M*_*Controls*_ = 25.0 ± 5.3; *p* = 0.26) and handedness (Fisher’s exact test (*N* = 52), *p* = 0.35) were not different between groups (for more details see Table 1). There were no differences between groups regarding smoking, drug use and medication (other than psychiatric medication) (in the following all calculated by using Fisher’s exact test (*N* = 52)): 26.9% (*N* = 7) of the PGAD patients and 19.2% (*N* = 5) of the controls were smokers (*p* = 0.74). Drug use occurred in 3.8% (*N* = 1) of both PGAD patients and controls (*p* = 1.00). Over-the-counter drugs such as vitamin B/C/D, iron, folic acid, zinc, calcium, and magnesium were taken by 53.8% PGAD patients (*N* = 14) and 30.8% of the controls (*N* = 8) (*p* = 0.16). Oral contraception was used by 30.8% (*N* = 8) PGAD patients and controls respectively (*p* = 1.00). L-thyroxine was taken by 19.2% (*N* = 5) PGAD patients and by 15.4% (*N* = 4) of the controls (*p* = 1.00). Antihypertensives were taken by 7.7% (*N* = 2) PGAD patients and by 15.4% (*N* = 4) controls (*p* = 0.67). Statins and antiplatelet drugs were taken by 3.8% (*N* = 1) of the PGAD patients and control subjects each (*p* = 1.00). Antidiabetics were taken by 3.8% (*N* = 1) of the PGAD patients and by none of the controls (*p* = 1.00). Urologicals and H1-receptor-blockers, proton pump inhibitors and beta-2-agonists were each taken by 7.7% (*N* = 2) PGAD patients and by none of the controls (*p* = 0.49).

**Table 1:** Sociodemographic, physical, cognitive, and family factors.

Variable	Group				Statistic
	PGAD Group (*n* = 26)		Control Group (*n* = 26)		*t-/p*-value (df)
	%	M ± SD/n	%	M ± SD/n	
**Demographic**					
Age		39.5 ± 14.7		39.5 ± 14.2	*0.0*/1.00 (50)^+^
Years of school education		11.9 ± 1.4		12.1 ± 1.4	*0.4*/0.70 (50)^+^
Employment status					0.49
Unemployed	7.7	2	3.8	1	
In training	15.4	4	26.9	7	
Retired	15.4	4	3.8	1	
Employed	61.5	16	65.4	17	
**Physical**					
Body mass index (BMI)		23.5 ± 4.4		25.0 ± 5.3	*− 1.1*/0.26 (50)^+^
**Familial**					
Marital Status					0.72
Unmarried/single	46.2	12	57.7	15	
Married	42.3	11	34.6	9	
Divorced	11.5	3	7.7	2	
Children	50.0	1.1 ± 1.2	42.3	0.7 ± 0.9	0.78
Misscariages	11.5	0.2 ± 0.4	3.8	0.0 ± 0.2	0.17
Abortions	15.4	0.2 ± 0.4	23.1	0.3 ± 0.5	0.37
**Sexual orientation**					
Kinsey Scale					
Heterosexual	92.3	24	80.8	21	
Bisexual	7.7	2	11.5	3	
Homosexual	0.0	0	7.7	2	

*M* mean, *SD* standard deviation, *df* degrees of freedom, *PGAD* Persistent genital arousal disorder.

*Denotes significant differences at *p* < 0.05. ^+^ Indicates calculated independent t-tests with their t- and *p*-values and degrees of freedom (df). Single numbers in the statistic-column represent the *p*-value, calculated by using Fisher's exact test for dichotomous variables (*N* = 52).

t-values are in [italic].

### Vegetative, gynaecological & sexual characterisation of PGAD

#### Gynaecological and vegetative history

The average cycle length was one day longer in PGAD patients than in controls (*M*_*PGAD*_ = 28.5 ± 1.7 days; *M*_*Controls*_ = 27.5 ± 0.9 days; *p* = 0.03). In both groups, the mean age at menarche was not different between the groups (*M*_*PGAD*_ = 12.9 ± 1.6; *M*_*Controls*_ = 12.9 ± 1.6; *p* = 1.00). Six subjects of both groups were menopausal. Concerning menstrual problems, no group differences could be detected (*N*_*PGAD*_ = 10, *N*_*Controls*_ = 9; *p* = 1.00). Similarly, no differences could be identified in taking hormonal contraceptives (*N*_*PGAD*_ = 7, *N*_*Controls*_ = 9; *p* = 0.76) and sterility treatment (*N*_*PGAD*_ = 4, *N*_*Controls*_ = 0; *p* = 0.11). There was no group-difference in self-reported masturbarche (*M*_*PGAD*_ = 13.5 ± 5.1; *M*_*Controls*_ = 15.5 ± 9.8; *p* = 0.39). Regarding micturition the following group differences were identified: elevated rates for PGAD patients in dysuria (*N*_*PGAD*_ = 6, *N*_*Controls*_ = 0; *p* = 0.02) as well as in urinary urgency (*N*_*PGAD*_ = 19, *N*_*Controls*_ = 1; *p* = 0.00) and in urinary frequency (*N*_*PGAD*_ = 12, *N*_*Controls*_ = 3; *p* = 0.01) (see Table 2).

**Table 2:** Vegetative, gynaecological and sexual characteristics.

Variable	Group				Statistic
	PGAD group (*n* = 26)		Control group (*n* = 26)		*t-*/*p*-value (df)
	%	M ± SD / n	%	M ± SD / n	
Spontaneous swelling of the genitals	65.4	17	3.8	1	< 0.001*
Extraordinary lubrication	34.6	9	7.7	2	0.038*
Spontaneous orgasms	30.8	8	0.0	0	0.004*
Restless legs symptoms	30.8	8	0.0	0	0.004*
Dysuria	23.1	6	0.0	0	0.023*
Elevated urinary urgency	73.1	19	3.8	1	< 0.001*
Elevated urinary frequency	46.2	12	11.5	3	0.013*
Cycle length [days]		28.5 ± 1.7		27.5 ± 0.9	*− 2.3*/0.029* (32)^+^
Menarche [age in years]		12.9 ± 1.6		12.9 ± 1.6	*0.0*/1.000 (50)^+^
Menstrual problems	38.5	10	34.6	9	1.000
Hormonal contraception	26.9	7	34.6	9	0.764
Menopause	23.1	6	23.1	6	1.000
Sterility treatment	15.4	4	0.0	0	0.110
Masturbarche [age in years]		13.5 ± 5.1		15.5 ± 9.8	*− 0.9*/0.393 (47)^+^
Hypersexual disorder (Kafka criteria)	11.5	3	3.8	1	0.610

*M* mean, *SD* standard deviation, *df* degrees of freedom, *PGAD* persistent genital arousal disorder.

*Denotes significant differences at *p* < 0.05. ^+^ Indicates calculated independent t-tests with their t- and *p*-values and degrees of freedom (df). Single numbers in the statistic-column represent the *p*-value, calculated by using Fisher’s exact test for dichotomous variables (*N* = 52).

t-values are in [italic].

#### Sexual history and PGAD symptomatology

According to hypersexual disorder, no group differences were detected (*N*_*PGAD*_ = 3, *N*_*Controls*_ = 1; *p* = 0.61). Swelling of the genitals (*N*_*PGAD*_ = 17, *N*_*Controls*_ = 1) as well as an extraordinary lubrication (*N*_*PGAD*_ = 9, *N*_*Controls*_ = 2) and spontaneous orgasm (*N*_*PGAD*_ = 8, *N*_*Controls*_ = 0) showed significantly higher rates in PGAD patients than in controls (*p*_*Swelling*_ = 0.00, *p*_*Lubrication*_ = 0.04, *p*_*Orgasms*_ = 0.00). Significantly more diagnoses of restless legs syndrome were made in the PGAD group (*N*_*PGAD*_ = 8, *N*_*Controls*_ = 0; *p* = 0.00). Eight (30.8%) of the PGAD patients were diagnosed with a psychological/psychiatric diagnosis prior to the onset of the PGAD symptomatology. This included depression, anxiety disorders and obsessive–compulsive disorders. None of the subjects showed any abnormalities in the neurological examination (for more details see Table 2).

### PGAD-specific characteristics

The mean age at PGAD diagnosis was 34.7 ± 15.6 years, ranging between 11 and 66 years. PGAD symptomatology was present for a mean of *M* = 4.8 ± 5.2 years (*min* = 1, *max* = 20).

#### Symptom severity

On average, actual symptoms were estimated as 3.6 ± 1.9 on a VAS from 0–10. The most severe symptoms in the last 4 weeks averaged 6.8 ± 2.6 and the average symptom severity in the last 4 weeks amounted to an average of 4.9 ± 2.1.

#### Progression of symptoms

Half of the PGAD patients (13 of 26) stated to have continuous symptoms with slight fluctuations, 7 had continuous symptoms with symptom attacks, 4 had symptom attacks with symptom-free phases in between and 2 of them had symptom attacks with symptoms in between.

#### Localisation, extragenital manifestation and radiation

**Figure 3:**
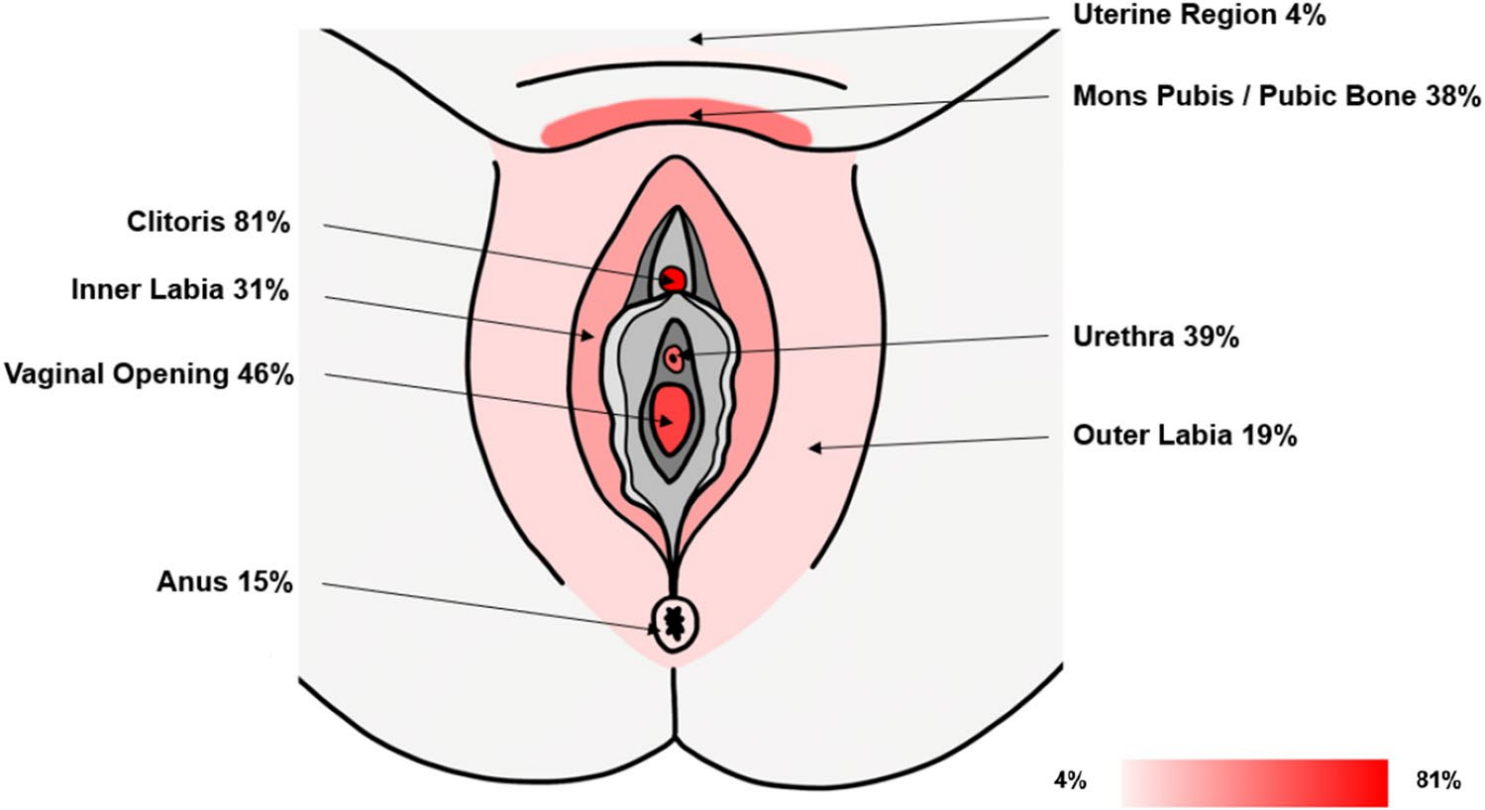
Localization of PGAD symptoms: Own illustration of a perineum in lithotomy position showing the localization of PGAD symptoms with their frequencies in percent. Multiple answers of each patient were possible.

**Figure 4:**
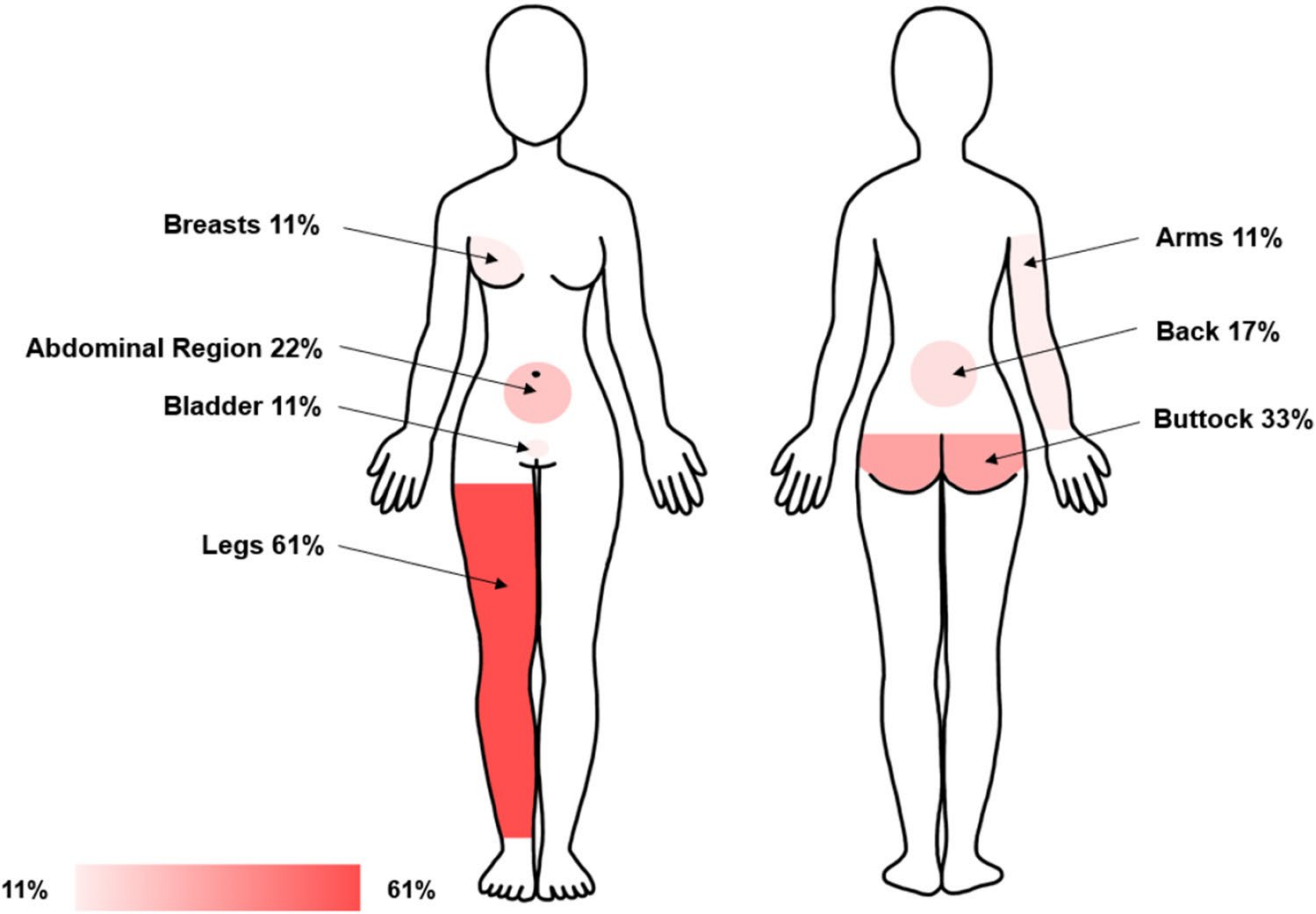
Radiation of PGAD symptoms: Own illustration of a schematic body of a woman showing the radiation of PGAD symptoms with their frequencies in percent. Multiple answers of each patient were possible. Sketch based on the following illustration: https://myloview.de/bild-weiblicher-korper-vorne-und-hinten-nr-65B021F.

Regarding the exact symptom localisation, 21 patients (80.8%) marked the clitoris, 13 (50.0%) the labia, 12 (46.2%) the vaginal opening, 10 (38.5%) the urethra, 5 each (19.2%) the mons pubis and pubic bone, 4 (15.4%) the anus, 1 (3.8%) the uterine region and none the ovarian region (see Fig. 3). 18 of 26 PGAD subjects (69.2%) reported a radiation to other regions of the body. 11 described a radiation in the legs, 6 in the direction of the buttocks, 4 in the abdominal region and 3 in the back. 2 of each group reported radiation to the arms, breasts and bladder (see Fig. 4).

#### Symptom quality

11 patients (42.3%) reported a strong or very strong tingling or prickling sensation. Just as many felt strong or very strong unpleasant sensations when the region was slightly touched for example by clothing or the bedspread. 8 PGAD subjects (30.8%) described the symptoms as electrifying. 7 patients (26.9%) experienced heat/warmth or cold as well as slight pressure, e.g. with the fingers, as symptomatic. 7 (26.9%) subjects felt a hardly or slight burning symptomatology. 2 (7.7%) subjects associated the symptomatology with numbness.

#### Trigger and relief factors

96.2% (25 of 26) PGAD patients reported specific trigger factors. The most frequent trigger factors turned out to be wearing tight clothing (42.3%), mental stress and tension (38.5%), driving a car, bus or bicycle (38.5%), lying down (30.8%), sexual intercourse (26.9%), vibration (26.9%) and sitting upright (26.9%). All of the 26 PGAD patients were able to report factors for symptom relief. This specifically included distraction (53.8%), relaxation (30.8%), physical exercise (26.9%), masturbation (23.1%) and swimming (23.1%). There was no significant correlation between masturbation or sexual intercourse as triggers and at the same time as relief factors (*r*_*masturbation*_ = − 0.04; *p*_*masturbation*_ = 0.86; *r*_*intercourse*_ = − 0.12; *p*_*intercourse*_ = 0.56; *N* = 26). For details of trigger and relief factors see Table 3.

**Table 3:**
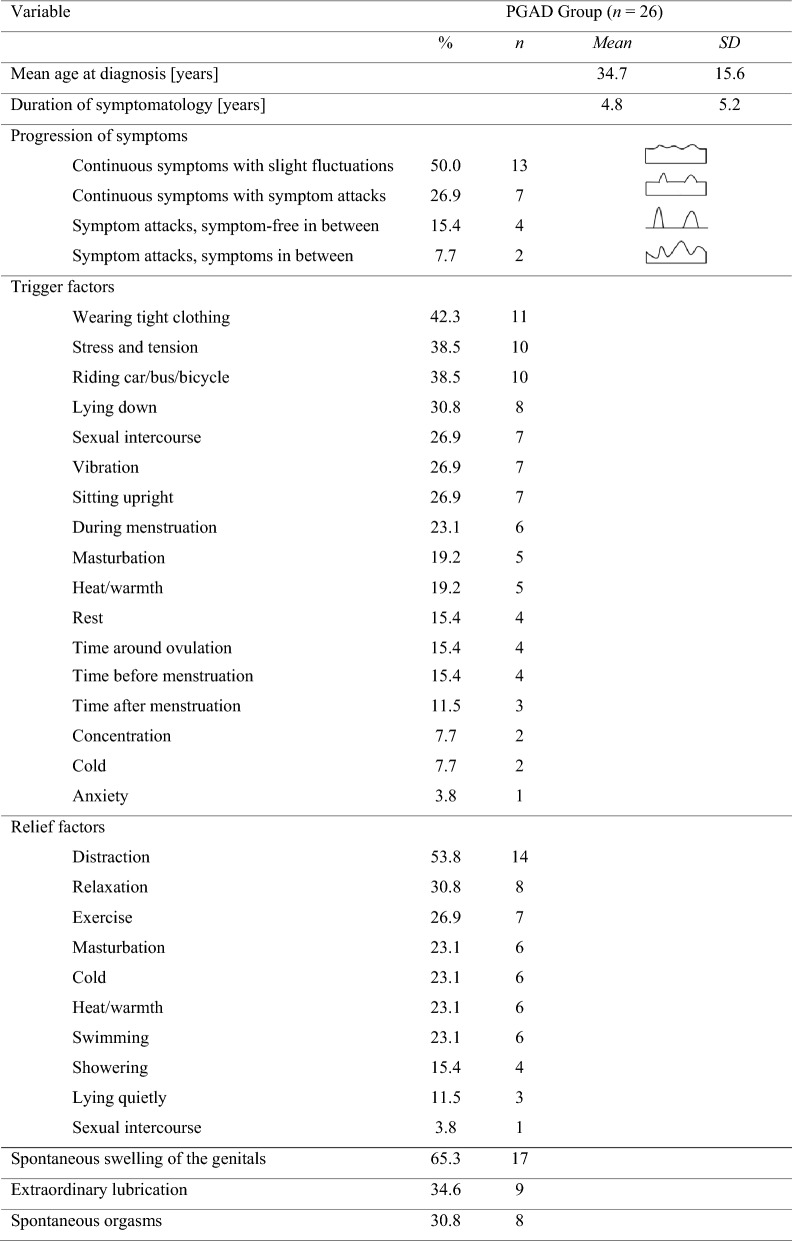
PGAD-related characteristics.

The sums of the main item radiation differ from the sums of the subitems, because the patients were able to make several statements regarding the individual subitems or had several diagnoses of the individual subitems. Swelling of the genitals, extraordinary lubrication and spontaneous orgasms were mentioned in Table 2 as well as in Table 3, because on the one hand the comparison with the control group was intended to be illustrated, and on the other hand the characterization of the symptomatology was intended to be highlighted by these three criteria.

*SD* standard deviation, *PGAD* persistent genital arousal disorder.

#### Onset of PGAD symptoms

19.2% reported an association of PGAD symptom onset with cessation of a selective serotonin reuptake inhibitor (SSRI) (*N* = 3) or an anticonvulsant (*N* = 2). The other subjects could not name an exact initiator for PGAD.

#### Previous treatment trials with drugs

18 (69.2%) PGAD subjects were taking medication for symptom relief while 8 (30.8%) of the 26 PGAD patients had not yet attempted PGAD medication at the time of the study. 14 subjects (77.8%) reported that their medication reduced their symptoms (Fisher’s exact test (*N* = 26), *p* = 0.00). Selective serotonin noradrenalin reuptake inhibitors (SNRI) and anticonvulsants were most commonly taken. More details on the subjective evaluation of the effect of the particular drugs can be found in Table 4.

**Table 4:** Medication relating PGAD symptomatology.

Drug	Provides relief	Does not provide relief	Total
	n	n	n
SNRI (Duloxetine, Milnacipran)	5	3	8
Anticonvulsants (Gabapentin, Pregabalin)	5	1	6
SSRI (Citalopram, Sertraline, Paroxetine)	2	2	4
Neuroleptics (Aripripazole, Clozapine)	2	0	2
NSMRI (Doxepine, Clomipramine)	2	0	2
Opioid (Oxycodone, Tramadol, Tilidine)	2	1	3
Benzodiazepine (Alprazolam)	1	0	1
	%	*n*	
PGAD associated with drug withdrawal	19.2	5	

*SNRI* selective serotonin noradrenalin reuptake inhibitor, *SSRI* selective serotonin reuptake inhibitor, *NSMRI* non selective monoamine reuptake inhibitors.

### Psychological features including psychiatric comorbidities and personality

The PGAD group showed significantly higher rates in sexual dysfunction (*N*_*PGAD*_ = 21, *N*_*Controls*_ = 1; *p* = 0.00). Significant group differences were found regarding lack or loss of sexual desire (*p* = 0.00), sexual aversion (*p* = 0.00), lack of sexual satisfaction (*p* = 0.01) and non-organic dyspareunia (*p* = 0.01). All PGAD patients reported development of sexual dysfunction after the onset of PGAD symptomatology. For more details see Table 5. Regarding sleep, the PSQI revealed decreased sleep quality in PGAD patients (PGAD = 80.8%, Controls = 46.2%, *p* = 0.02). Regarding quality of life, the PGAD group reported significantly lower scores in the WHO-QOL-Bref than the control-group. This concerned all subscales (physical health, social relationship, psychological health) except the environmental health domain (*t*_*overall*_(50) = 8.6, *p* = 0.00). No group differences could be detected on the CTQ subscales. Moreover, neither the PGAD patients nor the controls met the criteria for somatization disorder (for details see Table 5).

**Table 5:** Psychological characteristics and comorbidities.

Questionnaire subscales	Group				Statistic
	PGAD GROUP (*n* = 26)		Control group (*n* = 26)		*p*-value
	% / n	M ± SD	% / n	M ± SD	
Sexual dysfunction (F52)	80.8 / 21		3.8 / 1		<0.001*
Lack or loss of sexual desire (F52.0)	61.5 / 16		0.0 / 0		<0.001*
Sexual aversion (F52.10)	57.7 / 15		0.0 / 0		<0.001*
Lack of sexual satisfaction (F52.11)	38.5 / 10		3.8 / 1		0.005*
Failure of genital responses (F52.2)	19.2 / 5		0.0 / 0		0.051
Orgasmic disturbances (F52.3)	15.4 / 4		3.8 / 1		0.350
Non-organic vaginismus (F52.5)	11.5 / 3		0.0 / 0		0.235
Non-organic dyspareunia (F52.6)	26.9 / 7		0.0 / 0		0.010*
Increased sexual desire (F52.7)	11.5 / 3		0.0		0.235
Hospital Anxiety and Depression Scale					
Anxiety		9.2 ± 4.7		4.2 ± 2.8	<0.001*
Depression		7.3 ± 5.4		2.7 ± 2.9	<0.001*
Pittsburgh Sleep Quality Index	80.8 / 21		46.2 / 12		0.020*
WHO—quality of Life (Bref)					
Physical health		54.3 ± 22.5		85.4 ± 10.4	<0.001*
Social relationship		59.6 ± 18.2		77.6 ± 17.0	<0.001*
Psychological health		55.3 ± 19.1		76.6 ± 13.8	<0.001*
Environmental health		74.5 ± 15.2		81.6 ± 12.5	0.072
Childhood Trauma Questionnaire					
Emotional abuse		8.6 ± 4.4		7.4 ± 3.3	0.290
Physical abuse		5.7 ± 1.3		5.4 ± 1.1	0.257
Sexual abuse		5.7 ± 1.5		5.3 ± 1.1	0.344
Emotional neglect		10.0 ± 5.2		9.1 ± 5.0	0.534
Physical neglect		6.6 ± 2.2		7.0 ± 3.0	0.632

Group comparisons between PGAD and control group. The diagnoses of anxiety and depression were made with a cut off of 11 points or more with the Hospital Anxiety and Depression Scale (HADS) ^58^. Diagnosis of Anxiety: PGAD versus Controls = 30.8% versus 3.8%, *p* = 0.02*; Diagnosis of Depression: PGAD versus Controls = 23.1% vs. 3.8%, *p* = 0.10. *Denotes significant differences at *p* < 0.05. *p*-values were calculated by using independent t-tests or Fisher's exact test for dichotomous variables (*N* = 52). The sums of the main item Sexual dysfunction differ from the sums of the subitems, because one patient often showed several diagnoses of the individual subitems.

*M* mean, *SD* standard deviation, *PGAD* persistent genital arousal disorder.

The rates of all psychiatric comorbidities in axis I psychiatric disorders were higher in the PGAD group than in the control group (96.2% vs. 34.6%; *p* = 0.00). In PGAD patients, rates were significantly highest for previous major depression (N_*PGAD*_ = 22, *N*_*Controls*_ = 6; *p* = 0.00). Also, significantly higher rates for recurrent depression could be detected (*N*_*PGAD*_ = 9, *N*_*Controls*_ = 0; *p* = 0.00), but no differences could be detected regarding current depression (*N*_*PGAD*_ = 5; *N*_*Controls*_ = 0; *p* = 0.05). There were significantly more PGAD patients with agoraphobia (*N*_*PGAD*_ = 8, *N*_*Controls*_ = 0; *p* = 0.00) and generalized anxiety disorder (*N*_*PGAD*_ = 6, *N*_*Controls*_ = 0; *p* = 0.02). The rates of diagnoses in depression and anxiety found in the MINI were supported by the psychometric assessment of the Hospital Anxiety and Depression Scale: The scoring for a diagnosis of current depression was not significantly different between groups (*N*_*PGAD*_ = 6, *N*_*Controls*_ = 1; *p* = 0.10). Clearly higher scores of anxiety disorders could be detected in the PGAD group (*N*_*PGAD*_ = 8, *N*_*Controls*_ = 1; *p* = 0.02). There was a significantly higher rate of lifetime panic disorder in PGAD patients (*N*_*PGAD*_ = 9, *N*_*Controls*_ = 0; *p* = 0.00) but not a significant difference between groups in current panic disorder (*N*_*PGAD*_ = 4, *N*_*Controls*_ = 0; *p* = 0.11). Social anxiety disorder (*N*_*PGAD*_ = 2, *N*_*Controls*_ = 0; *p* = 0.49) occurred without any group differences, as well as obsessive–compulsive disorders (*N*_*PGAD*_ = 5, *N*_*Controls*_ = 2; *p* = 0.42). Regarding suicidality, no group differences could be detected (*N*_*PGAD*_ = 5, *N*_*Controls*_ = 2; *p* = 0.42). For alcohol use disorder of moderate or low severity no group differences could be detected (*N*_*PGAD*_ = 5, *N*_*Controls*_ = 3; *p* = 0.70). None had a high level of alcohol use disorder (see Tables 5 and 6 for all variables). As mentioned above, eight (30.8%) of the PGAD patients were diagnosed with a psychological/psychiatric diagnosis prior to the onset of the PGAD symptomatology. This included depression, anxiety disorders and obsessive–compulsive disorders. In 69.2% psychological comorbidities occurred after the diagnosis of PGAD.

**Table 6:** Main categories and findings regarding axis I and axis II (comorbid) disorders.

Disorder	Group				Statistic
	PGAD group (*n* = 26)		Control group (*n* = 26)		*p*-value
	n	%	n	%	
** Axis I disorders**					
Affective disorders	22	84.6	4	15.4	<0.001*
Current major depression	5	19.2	0	0.0	0.051
Recurrent major depression	9	34.6	0	0.0	0.002*
Previous major depression	22	84.6	6	23.1	<0.001*
Bipolar disorder (I or II)	1	3.8	0	0.0	1.000
Manic episode	1	3.8	0	0.0	1.000
Agoraphobia	8	30.8	0	0.0	0.004*
Generalized anxiety disorder	6	23.1	0	0.0	0.023*
Panic disorder					
Current panic disorder	4	15.4	0	0.0	0.110
Lifetime panic disorder	9	34.6	0	0.0	0.002*
Social anxiety disorder	2	7.7	0	0.0	0.490
Suicidality	5	19.2	2	7.7	0.420
Low	3	11.5	2	7.7	1.000
Moderate	0	0.0	0	0.0	–
High	2	7.7	0	0.0	0.490
Suicidal behavior disorder	1	3.8	0	0.0	1.000
Obsessive–compulsive disorder	5	19.2	2	7.7	0.419
Post-traumatic stress disorder	1	3.8	0	0.0	1.000
Alcohol use disorder	5	19.2	3	11.5	0.703
Substance use disorder	2	7.7	0	0.0	0.490
Any eating disorder	2	7.7	0	0.0	1.000
Somatization disorder	0	0.0	0	0.0	1.000
Psychiatric diagnosis prior to onset of PGAD symptomatology	7	26.9			
Psychiatric diagnosis after the onset of PGAD symptomatology	18	69.2			
**Axis II disorders**					
Any axis II disorder	20	76.9	18	69.2	0.755
Any cluster A disorder	5	19.2	3	11.5	0.703
Paranoid	4	15.4	1	3.8	0.350
Schizoid	0	0.0	1	3.8	1.000
Schizotypal	2	7.7	1	3.8	1.000
Any cluster B disorder	13	50.0	3	11.5	0.006*
Antisocial	4	15.4	3	11.5	1.000
Narcissistic	2	7.7	0	0.0	0.490
Borderline	6	23.1	1	3.8	0.099
Histrionic	1	3.8	1	3.8	1.000
Any cluster C disorder	15	57.7	18	69.2	0.565
Avoidant	8	30.8	3	11.5	0.173
Dependent	5	19.2	0	0.0	0.051
Obsessive–compulsive	11	42.5	16	61.5	0.267
Depressive	2	7.7	0	0.0	0.490
Negativistic (passive-aggressive)	4	15.4	0	0.0	0.110

Group comparisons between PGAD and control group.

*PGAD* Persistent Genital Arousal Disorder.

*Denotes significant differences at p < 0.05. *p*-values were calculated by using Fisher's exact test for dichotomous variables (*N* = 52). The sums of the main items differ from the sums of the subitems, because the same patient often showed several diagnoses of the individual subitems. The international neuropsychiatric interview for Axis I Disorders was used to determine the presence of psychiatric disorders. The Structured Clinical Interview for DSM-IV Axis II Disorders was used to determine comorbid personality disorders. Presence of a disorder was coded 1, absence was coded 0.

Regarding axis II psychiatric disorders, no group differences could be detected concerning cluster A and cluster C personality disorders (*p*_*ClusterA*_ = 0.70; *p*_*ClusterC*_ = 0.57). Rates of cluster B personality disorders were significantly higher in the PGAD group compared to the control group (*N*_*PGAD*_ = 13, *N*_*Controls*_ = 3; *p* = 0.01). 53.8% of the PGAD group fulfilled the DSM-IV criteria for two or more personality disorders, compared with 19.2% of controls (*t*(50) = -2.2, *p* = 0.03). For a detailed description of the individual clusters with their personality disorders, see Table 6.

### (Neuro-)Radiological, neurophysiological and gynaecological examination

None of the subjects showed any organic abnormality in the brain, except of an accidental finding of a benign tentorium meningioma in one control subject. Overall, 15 (57.7%) subjects from each of the two groups showed abnormalities on MRI in the spinal cord region, without any significant group differences (*p* = 1.00). The most notable and probably most relevant for PGAD were the fluid-filled nerve root cysts / Tarlov cysts. These occurred particularly at the sacral level of the spine (S1-S4) (except for one control, who also had one thoracic (Th12) and one lumbar (L2) nerve root cyst). There was no significant difference in the occurrence of these cysts between the two groups (*N*_*PGAD*_ = 13, *N*_*Controls*_ = 9; *p* = 0.26). Other abnormalities and their frequencies can be found in Table 7. Concerning abnormalities in the MRI of the pelvis, 24 (92.3%) PGAD patients and 23 (88.5%) controls showed abnormalities. No group differences could be detected (*p* = 1.00). In both groups, the diagnoses were most commonly ovarian cysts (*p* = 0.73), followed by venous convolutions (*p* = 0.78). Pelvic varices were a rare finding without any group differences (*N*_*PGAD*_ = 2, *N*_*Controls*_ = 0; *p* = 0.49). For more details see Table 7.

**Table 7:** Clinical findings of MRI.

MRI	Group				Statistic
	PGAD group (*n* = 26)		Control group (*n* = 26)		*p*-value
	n	%	n	%	
Brain	0	0.0	1	3.8	1.00
Pelvis	24	92.3	23	88.5	1.00
Ovarian cysts	22	84.6	20	76.9	0.73
Venous convolutions	14	53.8	12	46.2	0.78
Free fluid	6	23.1	9	34.6	0.54
Pelvic varices	2	7.7	0	0.0	0.49
Nabothian cysts	4	15.4	4	15.4	1.00
Spinal Cord	15	57.7	15	57.7	1.00
Nerve root cysts/Tarlov	13	50.0	9	34.6	0.26
Disc protrusion	6	23.1	6	23.1	1.00
Osteochondrosis	2	7.7	0	0.0	0.24
Sacroiliac joint effusion	1	3.8	0	0.0	0.49
Ventrolisthesis	1	3.8	0	0.0	0.49
Stenosis	1	3.8	1	3.8	1.00

Group comparisons between PGAD and control group.

*PGAD* persistent genital arousal disorder.

*Denotes significant differences at p < 0.05. *p*-values were calculated by Fisher’s exact test for dichotomous variables (*N* = 52). The sums of the main item Pelvis and Spinal Cord differ from the sums of the subitems, because the same patient often presented several abnormalities of the individual subitems.

The gynaecological examination showed no differences between the groups either (Fisher's exact test (*N* = 51), *p* = 0.11). One PGAD patient did not participate in the gynaecological examination. The abnormalities were mainly confined to the ovaries (for example polycystic ovarian syndrome).

21 PGAD patients and 24 controls participated in the neurophysiological examination. Abnormal examination findings could be identified in 8 PGAD patients and 3 controls (*p* = 0.08). 4 PGAD patients and none of the controls showed prolonged latencies in the SEP of the pudendal nerve (*M*_*PGAD*_ = 32.2 ± 6.1 ms; *M*_*Controls*_ = 30.5 ± 4.9 ms). No significant differences in the latencies in the group comparison were found (*p*_*1*_ = 0.35/*p*_*1*_ = 0.13). The amplitudes of the SEP of the pudendal nerve did also not differ significantly (*p* = 0.57), see Table 8. However, the occurrence of abnormal latencies (N1, P1) according to clinical assessment differed significantly from each other (Fisher’s exact test (*N* = 45), *p* = 0.04; t_1_(43) = − 5.192, *p*_1_ = 0.00; *t*_1_(43) = − 5.587, *p*_1_ = 0.00). Measurements on the tibial, peroneal, median and ulnar nerves were inconspicuous in both groups.

**Table 8:** Results of neurophysiology, pudendal nerve.

Neurophysiology	Group				Statistic
	PGAD group (*n* = 21)		Control group (*n* = 24)		*t-/p*-value (df)
	n	%	n	%	
Any abnormality in electroneurography	8	38.1	3	12.5	0.08

	Mean [ms]	SD	Mean [ms]	SD	
SEP Pudendal Nerve					
Latency N33	32.0	5.9	30.5	4.9	*− 0.95*/0.35 (43)^+^
Latency P40	38.6	5.9	36.1	5.0	*− 1.53*/0.13 (43)^+^
Amplitude N33/P40	1.0	0.5	0.9	0.6	*− 0.57*/0.57 (43)^+^

Group comparisons between PGAD and control group.

*df* degrees of freedom, *PGAD* persistent genital arousal disorder.

*Denotes significant differences at *p* < 0.05. ^+^ Indicates calculated independent t-tests with their t- and *p*-values and degrees of freedom (df). The single number in the statistic-column represents the *p*-value, calculated by using Fisher’s exact test for dichotomous variables (*N* = 45).

t-values are in [italic].

## Discussion

To our knowledge this is the first systematic and controlled analysis capturing vegetative, sexual and psychological features, psychiatric comorbidities as well as (neuro-)radiological, neurophysiological and gynaecological examinations in a group of 26 women suffering from PGAD and 26 healthy controls.

According to our data PGAD occurs in all age groups with a possible onset in every stage of life, ranging from late childhood to late adulthood. The average time from symptom onset to diagnosis of PGAD was 5 years, indicating that the disease is still unknown among most physicians. Typically, patients report relatively high and undulating genital arousal levels (~ 7 on a VAS) or continuous arousal with symptom attacks. We found no relation to symptoms of hypersexual disorder, however many subjects developed sexual dysfunctions after the onset of PGAD; most commonly lack of sexual desire, sexual aversion, and lack of sexual satisfaction was found.

On a phenomenological level, PGAD presents as a form of dysesthesia: Almost half of the patients stated that any contact of the genital area, e.g., through tight clothing, was perceived as an unpleasant and also tingling or prickling sensation. These sensations phenomenologically may fit to a small fibre sensory neuropathy of the pudendal nerve^6,12,20,22^, however the current neurophysiological examination did not indicate any pathological findings. Others reported triggering factors like prolonged sitting, lying down and any stimulation in the genital area (vibration, sexual intercourse, masturbation, bus riding, car driving and riding bicycle). These were discussed as a result of injury or entrapment of the pudendal nerve^17,18^. Likewise, the current study did not reveal any reports of traumatization or surgery in the pelvic/genital region. In this respect, the neurophysiological measurements were also inconspicuous except for prolonged SEPs of the pudendal nerve in four of the PGAD patients. Although this did not result in a statistical difference, attention should be paid to this in the future.

The localization of symptoms included the entire genital and pelvic floor region, with the clitoris being mentioned most frequently (81%), followed by the labia majora and minora (50%) and vaginal opening (46%). This finding is in line with a previous study^22^, showing hypersensitivity in the dermatomes of the dorsal branch of the pudendal nerve and the ilioinguinal nerve. Women in the current study also indicated symptoms of the urethra and the anal region, suggesting that not only the dorsal ramus of the pudendal nerve may be involved but also perineal branches. This would contradict Waldinger’s and colleagues’ assumption of an isolated neuropathy of the dorsal nerve as cause of PGAD.

Almost 70% of PGAD patients reported radiation to other parts of the body, especially to the legs, the buttocks and the abdominal region. Of note, Tarlov cysts can cause leg, buttock and lower back pain and are also associated with bladder dysfunctions. However, the current study did not show differences in the number of Tarlov cysts between the groups (50% vs. 35%). In single cases PGAD associated sacral cysts were successfully treated by neurosurgical resection^59^ or nerve blocks with epidural anaesthesia^18^, although long term outcomes still need to be assessed in these studies. Herniated discs were also rare in women with PGAD and equally frequent in the controls, which also weakens the hypothesis of a radiculopathy. Other abnormalities such as osteochondrosis, sacroiliac joint effusions, ventrolisthesis and spinal stenosis occurred only sporadically and have no meaningful value.

Heat has also been described as a trigger factor, which could be explained by the increased genital blood flow due to the dilation of the vessels by the heat, resulting in a possible higher awareness of genital sensation. Thus, some patients also indicated that cool packs, cold showers, and swimming would help to provide relief. However, more than 20% of the patients also stated that heat would also provide relief, which contradicts the statement above.

Besides modulation of blood circulation and the accompanying chance in awareness by heat, also medication can influence PGAD symptoms. As mentioned above, the onset of PGAD as a discontinuation phenomenon has so far only been reported for drugs with a serotonergic component^2,24,60–64^. The connection with serotonergic drugs may be explained as follows: When taking an SSRI, serotonin suppresses the production of atrial natriuretic peptide (ANP) in the atrium. If the SSRI is now withdrawn, more ANP is released, which leads to vasodilation and to a return to baseline libido. Consequently, this results in increased genital blood flow and possible newfound awareness of genital sensations^20,65^. Also, a release of the brake of the central nervous system could lead to neuronal hyperexcitability^32^. This discontinuation phenomenon occurred in 5 of the 26 PGAD patients, 3 of whom discontinued an SSRI and 2 of whom reported the phenomenon after discontinuing an anticonvulsant (see also^32^). Anticonvulsants cause a decrease in neuronal activity, in particular a decrease in neuronal excitation and an increase in neuronal inhibition^66^. Cessation of the anticonvulsant could also result in a rebound effect. Most of the patients treated with medication were taking an SNRI or anticonvulsant. The majority of them also reported symptom relief from medication presumably via the modes of action described above. SSRIs, neuroleptics, non-selective monoamine reuptake inhibitors (NSMRIs), opioids, and benzodiazepines were also taken in exceptional cases. However, the evidence of the efficacy of the drugs is not indicative with such a small number. In summary, the potential benefits of the drugs have not yet been captured widely enough. More accurate data collection on the medication used before the onset of PGAD and on the treatment of PGAD should be considered in future studies.

In summary, a number of trigger and relief factors were reported and the trigger for one may even provide relief for the other (e.g., sexual intercourse, lying down, heat/warmth). Thus, the high variability of trigger and relief factors suggests different causes for PGAD, with both explanations in the direction of peripheral and central neurological deficits. Whereas peripheral stimuli with a central origin could also worsen symptoms. Trigger factors are usually accompanied by the activation of mechanoreceptors (pressure and vibration) as well as psychological factors and menstrual cycle (see below).

Remarkably, more than 30% of the PGAD women compared to none controls reported restless legs symptoms, supporting the hypothesis that PGAD may be a variant of RLS and also of overactive bladder syndrome (OAB). Interestingly both syndromes are discussed in terms of a hyperexcitability of the dopaminergic system^26^. The possible relationship of the syndromes and the associated dopamine deficiency could be one reason for the significantly increased rates of urinary urgency and urinary frequency in PGAD women. Another reason for this finding might be the overlap of bladder and genital function with regard to its spinal representation (S2-S4). Nevertheless, there are barely reports on successful and sustained relief of PGAD symptoms by L-Dopa or dopamine agonists.

With respect to other discussed reasons of PGAD pelvic varices and venous convolutes were found equally seldom in both groups. Waldinger et al.^21^ highlighted the importance of an age-matched control group especially on the prevalence of pelvic varices. For example, Thorne and Stuckey^67^ reported a case with pelvic congestion syndrome. In this case, coil embolization as treatment could lead to a reduction of PGAD symptoms. In fact, there was one patient in the sample of the iPGAD study who had already benefited from a previous coil embolization, but this did not lead to a complete remission. This, together with the equal distribution of varices and cysts, confirms the suspicion that pelvic varices or pelvic congestion syndrome may be a cause in single cases but not be considered as a general etiological factor.

From differential diagnostic levels there was no evidence for elevated levels of somatization disorders as a reason for genital symptoms as found in PGAD. Apart from this, current depressive episode or current panic disorder were equally prevalent in both groups. In contrast, significantly more PGAD patients suffered from recurrent or previous depressive episode and lifetime panic disorders, with most of them starting after the onset of PGAD symptoms (7 of 26 PGAD patients had depression prior to PGAD, but in 18 patients’ depression occurred after the onset of PGAD), suggesting that PGAD has a negative impact on the patient’s mental health instead of the reverse (depression as a cause of PGAD). The significantly higher incidence of agoraphobia and generalized anxiety disorder in the PGAD group could also result from persistent sexual arousal/agitation as they fear that someone might detect their genital arousal. The patients no longer dare to be around people and large areas, a constant apprehension and tension as well as free-floating fears can be aggravated by the persistent agitation as explained in the fear avoidance model by Jackowich and Pukall^12^. If there is an arousal experience, the patients start to interpret the arousal as alarming and begin to catastrophize. This leads to arousal-related fear and to an avoiding of behaviours related to the arousal. This results in increased hypervigilance to the arousal. The attempt to avoid the hypervigilant arousal eventually leads to negative psychosocial outcomes like depression, anxiety and dysfunctional social interaction. This in turn increases the awareness of the arousal and allows the vicious circle to continue^12,40^. The model is also consistent with the finding that more than 50% of the PGAD patients experienced symptom relief through distraction and symptoms were triggered by stress which leads to elevated levels of vigilance.

PGAD has a tremendous impact on quality of life as indicated by WHO-QOL-BREF measures and also sleep quality was reduced significantly. No differences were found regarding other psychological comorbidities such as alcohol disorders, obsessive–compulsive disorders, social anxiety, eating disorders and PTSD. Particularly noteworthy is the lack of significant difference between the groups regarding suicidality, which has been frequently postulated, e.g., by^10,40,42,43^. The assumption that PGAD arises from trauma like sexual abuse could not be supported by the childhood trauma questionnaire (CTQ) nor the psychiatric history. A questionnaire on sexual trauma after childhood was not explicitly included in the examinations. However, a detailed sexual history was taken, and—according to clinical experience—a possible severe sexual trauma would have been reported. Also, as mentioned above, PTSD was not an issue in both groups. No abnormalities in personality disorders could be identified in the PGAD patients as no differences were found in cluster A and C disorders. However, cluster B disorders occurred significantly more often in PGAD patients, but the individual personality disorders that fall under cluster B did not differ. Overall, the mental constitution of the patients was hardly different from that of the control group. Thus, we assume that PGAD patients are psychologically healthy, except for the mental burden resulting from PGAD.

Since PGAD is a rare disease, a reasonably good number of subjects have already been recruited in the iPGAD study. However, an even larger sample in a design with healthy controls is needed to further corroborate the presented findings, especially regarding the neurophysiological data, and the use of medication. It remained partly unclear to what extent a somatoform autonomic dysfunction (F45.34) could be present in the patients. For this purpose, a group with diagnosed somatoform autonomic dysfunctions should have been included in the comparisons. Also, for more detailed comparisons, with regard to the theory of (functional) nerve lesions, a group of patients should have been included who were treated surgically or who could achieve an improvement through physical therapy. The distinction between PGAD as being a consequence or a cause of mental illness (especially personality disorders and also general anxiety disorder and agoraphobia) remains partially unclear and needs to be examined in more detail. The most likely interpretation is towards a consequence of PGAD. PGAD presents as a heterogeneous syndrome and seems to have multiple triggers. In summary, there was no evidence of a causal relationship to a specific finding as it has been previously discussed. However, further investigations in a larger sample and additional techniques are needed to find out how and where the peripheral or central nervous systems are involved. Regarding clinical implications, health care professionals (in particular general practitioners, psychiatrists, psychotherapists, gynaecologists, urologists and neurologists) should be aware of PGAD symptoms. PGAD is a rare—but possibly highly underreported—and serious disease that can be recognized by already assessing the main symptoms of PGAD as well as triggering and relieving factors. This at least allows symptomatic therapy to be initiated quickly by using combinations of physiotherapy, psychopharmacotherapy, and/or psychotherapy. In addition, psychiatric comorbidities and, where appropriate, quality of life and sleep quality should be taken into account.

### Supplementary Information

Supplementary Information.

## Data Availability

The datasets generated during and/or analysed during the current study are available from the corresponding author on reasonable request.

## References

[1] Leiblum SR (2003). Arousal disorders in women: complaints and complexities. Med. J. Aust..

[2] Jackowich R, Pink L, Gordon A, Poirier É, Pukall CF (2018). Symptom characteristics and medical history of an online sample of women who experience symptoms of persistent genital arousal. J. Sex Marital Ther..

[3] Kamatchi R, Ashley-Smith A (2013). Persistent genital arousal disorder in a male: A case report and analysis of the cause. BJMP.

[4] Kruger TH, Hartmann U (2016). A case of comorbid persistent genital arousal disorder and premature ejaculation: Killing two birds with one stone. J. Sex Marital Ther..

[5] Stevenson, B. J. & Köhler, T. S. First reported case of isolated persistent genital arousal disorder in a male. *Case Rep. Urol.* **2015**, (2015). 10.1155/2015/465748 PMC4342067 25767735

[6] Waldinger MD, Venema PL, van Gils AP, de Lint GJ, Schweitzer DH (2011). Stronger evidence for small fiber sensory neuropathy in restless genital syndrome: two case reports in males. J. Sex. Med..

[7] Leiblum S (2001). Persistent sexual arousal syndrome: A newly discovered pattern of female sexuality. J. Sex Marital Ther..

[8] Goldstein I (2021). International society for the study of women’s sexual health (ISSWSH) review of epidemiology and pathophysiology, and a consensus nomenclature and process of care for the management of persistent genital arousal disorder/genito-pelvic dysesthesia (PGAD/GPD). J. Sex. Med..

[9] Parish SJ (2016). Toward a more evidence-based nosology and nomenclature for female sexual dysfunctions: part II. J. Sex. Med..

[10] Gündüz N, Polat A, Turan H (2019). Persistent genital arousal disorder treated with duloxetine: A case report. Turk Psikiyatri Derg..

[11] Jackowich RA, Pink L, Gordon A, Pukall CF (2016). Persistent genital arousal disorder: A review of its conceptualizations, potential origins, impact, and treatment. Sex. Med. Rev..

[12] Jackowich RA, Pukall CF (2020). Persistent genital arousal disorder: A biopsychosocial framework. Curr. Sex. Health Rep..

[13] Leiblum S (2006). Persistent genital arousal disorder: What it is and what it isn’t. Contemp Sex.

[14] Leiblum S, Brown C, Wan J, Rawlinson L (2005). Persistent sexual arousal syndrome: A descriptive study. J. Sex. Med..

[15] Bedell S, Goldstein AT, Burrows L (2014). A periclitoral mass as a cause of persistent genital arousal disorder. J. Sex. Med..

[16] Cohen SD (2017). Diagnosis and treatment of persistent genital arousal disorder. Rev. Urol..

[17] Klifto K, Dellon AL (2020). Persistent genital arousal disorder: Treatment by neurolysis of dorsal branch of pudendal nerve. Microsurgery.

[18] Klifto KM, Dellon AL (2020). Persistent genital arousal disorder: Review of pertinent peripheral nerves. Sex. Med. Rev..

[19] Aumüller, G., Engele, J., Kirsch, J. & Mense, S. Duale Reihe Anatomie (3., Auflage). *DUALE REIHE Hrsg. Von Alexander Bob Konstantin Bob* (2014).

[20] Facelle TM, Sadeghi-Nejad H, Goldmeier D (2013). Persistent genital arousal disorder: Characterization, etiology, and management. J. Sex. Med..

[21] Waldinger MD, Van Gils AP, Ottervanger HP, Vandenbroucke WV, Tavy DL (2009). Persistent genital arousal disorder in 18 Dutch women: Part I. MRI, EEG, and transvaginal ultrasonography investigations. J. Sex. Med..

[22] Waldinger MD, Venema PL, Van Gils AP, Schweitzer DH (2009). New insights into restless genital syndrome: static mechanical hyperesthesia and neuropathy of the nervus dorsalis clitoridis. J. Sex. Med..

[23] Oaklander AL, Sharma S, Kessler K, Price BH (2020). Persistent genital arousal disorder: a special sense neuropathy. Pain Rep..

[24] de Magalhães FJC, Kumar MT (2015). Persistent genital arousal disorder following selective serotonin reuptake inhibitor cessation. J. Clin. Psychopharmacol..

[25] Giraldi A, Rellini AH, Pfaus J, Laan E (2013). Female Sexual arousal disorders. J. Sex. Med..

[26] Sforza E, Hupin D, Roche F (2017). Restless genital syndrome: Differential diagnosis and treatment with pramipexole. J. Clin. Sleep Med..

[27] Waldinger MD, Schweitzer DH (2009). Persistent genital arousal disorder in 18 Dutch women: Part II—A syndrome clustered with restless legs and overactive bladder. J. Sex. Med..

[28] Anzellotti F (2010). Persistent genital arousal disorder associated with functional hyperconnectivity of an epileptic focus. Neuroscience.

[29] Aull-Watschinger S, Pataraia E, Baumgartner C (2008). Sexual auras: Predominance of epileptic activity within the mesial temporal lobe. Epilepsy Behav..

[30] Reading PJ, Will RG (1997). Unwelcome orgasms. The Lancet.

[31] Goldstein, I., De, E. J. B. & Johnson, J. Persistent sexual arousal syndrome and clitoral priapism. *Women’s Sex. Funct. Dysfunct. Study Diagn. Treat. Lond. Taylor Francis* 674–85 (2006).

[32] Kruger TH (2018). Can pharmacotherapy help persistent genital arousal disorder?. Expert Opin. Pharmacother..

[33] Kruger TH, Schippert C, Meyer B (2020). The pharmacotherapy of persistent genital arousal disorder. Curr. Sex. Health Rep..

[34] Battaglia C, Venturoli S (2009). Persistent genital arousal disorder and trazodone. Morphometric and vascular modifications of the clitoris. A case report. J. Sex. Med..

[35] Berk M, Acton M (1997). Citalopram-associated clitoral priapism: A case series. Int. Clin. Psychopharmacol..

[36] Calabrò RS (2017). Lamotrigine-induced persistent genital arousal disorder: An unusual side effect. Epilepsy Behav..

[37] Healy D, Le Noury J, Mangin D (2018). Enduring sexual dysfunction after treatment with antidepressants, 5 α-reductase inhibitors and isotretinoin: 300 cases. Int. J. Risk Saf. Med..

[38] Mahoney S, Zarate C (2007). Persistent sexual arousal syndrome: A case report and review of the literature. J. Sex Marital Ther..

[39] Gadit A (2013). Persistent genital arousal disorder: A clinical challenge. Case Rep..

[40] Jackowich RA, Poirier É, Pukall CF (2020). A comparison of medical comorbidities, psychosocial, and sexual well-being in an online cross-sectional sample of women experiencing persistent genital arousal symptoms and a control group. J. Sex. Med..

[41] Leiblum SR, Chivers ML (2007). Normal and persistent genital arousal in women: New perspectives. J. Sex Marital Ther..

[42] Goldmeier D, Leiblum SR (2006). Persistent genital arousal in women: A new syndrome entity. Int. J. STD AIDS.

[43] Korda JB, Pfaus JG, Kellner CH, Goldstein I (2009). Persistent genital arousal disorder (PGAD): Case report of long-term symptomatic management with electroconvulsive therapy. J. Sex. Med..

[44] Leiblum S, Seehuus M, Goldmeier D, Brown C (2007). Psychological, medical, and pharmacological correlates of persistent genital arousal disorder. J. Sex. Med..

[45] Pernot-Masson AC (2020). Persistent genital arousal disorder: A neurodevelopmental hypothesis. Eur. J. Trauma Dissociation.

[46] Pink L, Rancourt V, Gordon A (2014). Persistent genital arousal in women with pelvic and genital pain. J. Obstet. Gynaecol. Can..

[47] Leiblum R, Sandra SGN (2001). Persistent sexual arousal syndrome: A newly discovered pattern of female sexuality. J. Sex Marital Ther..

[48] Kinsey, A. C., Pomery, W. B. & Martin, C. E. Kinsey Scale. *Pers. Soc. Psychol. Bull.* (1948).

[49] Kafka, M. P. The paraphilia-related disorders. *Princ. Pract. Sex Ther.* 471–503 (2000).

[50] Freynhagen R, Baron R, Gockel U, Tölle TR (2006). pain *DETECT* : a new screening questionnaire to identify neuropathic components in patients with back pain. Curr. Med. Res. Opin..

[51] Rief W, Hiller W, Heuser J (2008). Screening für somatoforme Störungen (SOMS).

[52] Lecrubier Y (1997). The Mini International Neuropsychiatric Interview (MINI). A short diagnostic structured interview: Reliability and validity according to the CIDI. Eur. Psychiatry.

[53] First, M. B. & Gibbon, M. The structured clinical interview for DSM-IV axis I disorders (SCID-I) and the structured clinical interview for DSM-IV axis II disorders (SCID-II). (2004).

[54] Zigmond AS, Snaith RP (1983). The hospital anxiety and depression scale. Acta Psychiatr. Scand..

[55] Buysse DJ, Reynolds CF, Monk TH, Berman SR, Kupfer DJ (1989). The Pittsburgh sleep quality index: A new instrument for psychiatric practice and research. Psychiatry Res..

[56] WHOQOL Group (1998). Development of the World Health Organization WHOQOL-BREF quality of life assessment. Psychol. Med..

[57] Bernstein DP, Fink L, Handelsman L, Foote J (1998). Childhood trauma questionnaire. Assess. Fam. Violence Handb. Res. Pract..

[58] Brehaut E (2020). Depression prevalence using the HADS-D compared to SCID major depression classification: An individual participant data meta-analysis. J. Psychosom. Res..

[59] Feigenbaum F, Boone K (2015). Persistent genital arousal disorder caused by spinal meningeal cysts in the sacrum: Successful neurosurgical treatment. Obstet. Gynecol..

[60] Eibye, S. & Jensen, H. M. Persistent genital arousal disorder: confluent patient history of agitated depression, paroxetine cessation, and a tarlov cyst. *Case Rep. Psychiatry* **2014**, (2014). 10.1155/2014/529052 PMC4265540 25525548

[61] Freed L (2005). Letters to the editor: Persistent sexual arousal syndrome. J. Sex. Med..

[62] Leiblum SR, Goldmeier D (2008). Persistent genital arousal disorder in women: case reports of association with anti-depressant usage and withdrawal. J. Sex Marital Ther..

[63] Miyake K (2018). Restless genital syndrome induced by Milnacipran. Clin. Neuropharmacol..

[64] Philippsohn S, Kruger TH (2012). Persistent genital arousal disorder: successful treatment with duloxetine and pregabalin in two cases. J. Sex. Med..

[65] Goldmeier D, Bell C, Richardson D (2006). Withdrawal of selective serotonin reuptake inhibitors (SSRIs) may cause increased atrial natriuretic peptide (ANP) and persistent sexual arousal in women?. J. Sex. Med..

[66] Lüllmann H, Mohr K, Hein L (2003). Pharmakologie und toxikologie. 15. Auflage. Georg Thieme Verl. Stuttgart.

[67] Thorne C, Stuckey B (2008). CASE REPORT: Pelvic congestion syndrome presenting as persistent genital arousal: a case report. J. Sex. Med..

