# Supplementary material for: Clinical characterisation of women with persistent genital arousal disorder: the iPGAD-study
Source: Sci Rep. 2023 Dec 20;13:22814. doi: 10.1038/s41598-023-48790-2 (PMC10739833; doi:10.1038/s41598-023-48790-2)
Supplement: Supplementary file 1 — Supplementary Information. [file 41598_2023_48790_MOESM1_ESM.pdf]

Supplements

Supplement 1. Imaging Parameters (MRI)

Siemens	FOV (mm)	Layer thickness (mm)	TR (ms)	TE (ms)	Flip Angle (deg)	Distance Factor (%)
T1, sagittal brain	256x240	1.0	5000.0	2.98	4	50
T2, sagittal conus	300x300	3.0	3500.0	91.0	160	10
T2, transversal conus	240x240	4.0	7770.0	41.0	130	10
T2, transversal sacrum	240x240	4.0	7770.0	41.0	130	10
T2, transversal pelvis	380x308	5.0	8320.0	99.0	140	10
T2, coronal pelvis	380x380	5.0	5020.0	122	120	10
T2, transversal pelvis	480x330	5.0	7880.0	57.0	156	10
T1, transversal pelvis	200x200	3.0	854.0	12.0	140	10

Notes: Abbreviations: FOV = Field of View, TR = repetition time, TE = echo time, deg = degree.
